# *Citrus bergamia* Juice Extract Attenuates **β**-Amyloid-Induced Pro-Inflammatory Activation of THP-1 Cells Through MAPK and AP-1 Pathways

**DOI:** 10.1038/srep20809

**Published:** 2016-02-08

**Authors:** Monica Currò, Roberto Risitano, Nadia Ferlazzo, Santa Cirmi, Chiara Gangemi, Daniela Caccamo, Riccardo Ientile, Michele Navarra

**Affiliations:** 1 Department of Biomedical Sciences and Morphological and Functional Images, University of Messina, Messina, I-98100, Italy; 2 Department of Chemical, Biological, Pharmaceutical and Environmental Sciences, University of Messina, Messina, I-98168, Italy

## Abstract

Flavonoids have been shown to be effective in protecting against age-related cognitive and motor decline in both *in vitro* and *in vivo* models. Recently, a flavonoid-rich extract of *Citrus bergamia* juice (BJe) has been shown to display anti-oxidant and anti-inflammatory properties against LPS-induced activation of human THP-1 monocytes. In the light of these observations, we wondered whether BJe may be beneficial against neuroinflammatory processes, such as those observed in Alzheimer’s disease. To this aim we used THP-1 monocytes to investigate the mechanisms underlying the beneficial potential of BJe against amyloid-beta_1–42_ (Aβ_1−42_) -mediated inflammation. Exposure of THP-1 cells to Aβ_1−42_ significantly induced the expression and secretion of IL-6 and IL-1β in THP-1 cells and increased the phosphorylation of ERK 1/2 as well as p46 and p54 members of JNK family. Moreover, Aβ_1−42_ raises AP-1 DNA binding activity in THP-1-treated cells. Interestingly, all these effects were reduced in the presence of BJe. Our data indicate that BJe may effectively counteract the pro-inflammatory activation of monocytes/microglial cells exposed to amyloid fibrils, suggesting a promising role as a natural drug against neuroinflammatory processes.

In recent years, much attention has been paid to the neuroprotective effects of flavonoids, which have been shown to be effective in protecting against both age-related cognitive and motor decline *in vivo*. Their potential health benefit is due to both the antioxidant properties and the ability to cross the blood-brain barrier, thereby modulating a number of intracellular signaling pathways responsible for important physiological activities within the brain, including regulation of cell survival/apoptotic genes and mitochondrial function^1^,^2^. As a consequence, flavonoids have been suggested as novel therapeutic agents for the reduction of the deleterious effects of neuroinflammation in the brain as well as potential preventive drugs for neurodegenerative disease development^3^.

*Citrus bergamia* Risso & Poiteau (bergamot) is an endemic plant of the Calabria region (Italy) cultivated along the southern coast. It has long been used for the extraction of its essential oil from the fruit peel, mainly used in both perfume industry and aromatherapy^4^, and lately investigated for its anticancer^5^,^6^ and neuroprotective effects^7^. Bergamot juice (BJ), obtained by squeezing the endocarp of the fruits, has been considered for long time just a byproduct, until different studies revealed its beneficial effect on human health. In this regard, we recently demonstrated that BJ reduced signaling pathways related to proliferation, adhesion and migration of cancer cells, both *in vitro*^8^,^9^ and *in vivo*^10^. Moreover, we documented that its antiproliferative effect was exerted by the flavonoid fraction (BJe), which is able to reduced proliferation of human colon cancer cells by inducing apoptosis^11^. Interestingly, BJe has been recently shown to counteract effectively the LPS-induced inflammatory activation of human THP-1 monocytes^12^, that are considered a good model of human monocyte/macrophages due to their well characterized ability to secrete inflammatory mediators after stimulation by several agents^13^. The anti-inflammatory activity of BJe was also demonstrated in an *in vivo* model^14^, suggesting a possible role in treating inflammatory processes because its favorable balance between safety and efficacy^15^. Finally, very recently BJe has shown its potential as antioxidant^16^ and antimicrobial agent^17^.

Clear evidence demonstrates that the mechanisms responsible for the transduction and amplification of inflammatory responses contribute to the development of neurotoxicity. Hallmarks of chronic inflamed tissues are the presence of an increased number of monocytes, as well as monocyte-derived tissue macrophages, that can be referred to microglial cells in the central nervous system (CNS)^18^. Chronic immune activation, triggered by different stimuli, can be considered a common feature of chronic neurodegenerative disorders, including Alzheimer’s disease (AD) and Parkinson’s disease (PD). AD is characterized by the presence of reactive microglia around senile plaques, abundant intracellular neurofibrillary tangles, and progressive loss of neurons in the brains of affected patients^19^. The plaques are primarily composed of amyloid-β (Aβ) peptide fibrils assembled by non-covalent polymerization of Aβ monomers that derive from the enzymatic cleavage of amyloid precursor protein (APP)^20^. Noteworthy, Aβ peptides drive cerebral neuroinflammation by activating microglia and astrocytes, which in turn promote the expression of inflammatory cytokines, the activation of the complement cascade, and the induction of inflammatory enzyme systems^21^.

The accumulation of Aβ is thought to be an early and perhaps necessary feature of AD^19^. The predominant forms of Aβ are the (1–40) and (1–42) fragments. These latter are the major constituent of senile plaques and are present in minor amounts in the circulation^22^. In AD, the presence of monocytes/macrophages in the blood vessel walls and activated microglial cells in the brain parenchyma has been associated with increased deposition of Aβ within the brain^23^. However, there is evidence that Aβ deposition initiates a microglia-mediated inflammatory response that culminates in neuronal loss and cognitive decline in AD^24^.

Given that flavonoids were shown to display protective effects against both pro-oxidant and inflammatory stimuli, in this study we evaluated the ability of BJe to modulate Aβ_1–42_-mediated pro-inflammatory activation of THP-1 monocytes.

## Results

**Figure 1:**
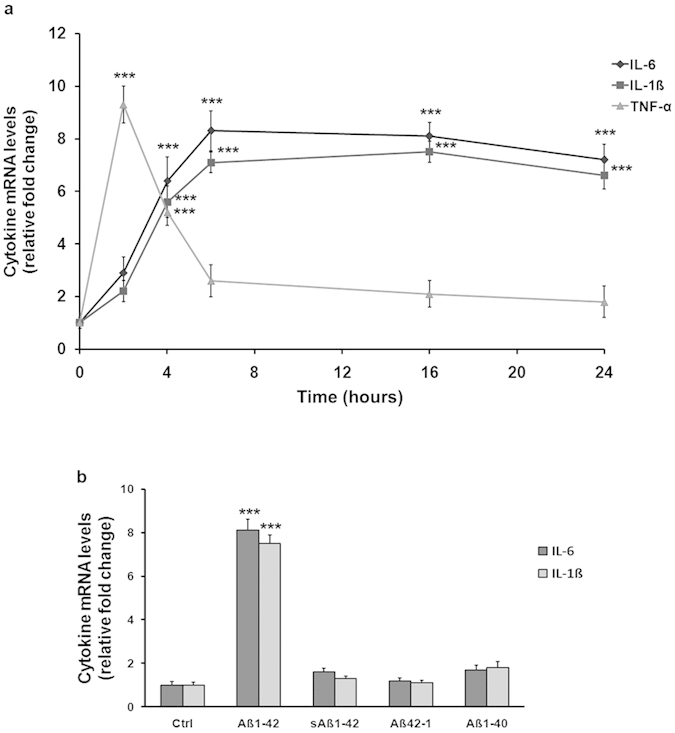
Cytokine gene expression in THP-1 monocytes exposed to different amyloid peptides. (**a**) The time course of gene expression for IL-6, IL-1β and TNF-α following incubation with 0.5 μM Aβ_1−42_ in THP-1 cells over a 24 h period is shown. (**b**) Effects of Aβ_1−42_, sAβ_1−42_, Aβ_42−1_ and Aβ_1−40_ on cytokine mRNA levels of both IL-6 and IL-1β in THP-1 cells after 16 h of incubation. Results from real-time PCR are expressed as relative fold change of mRNA levels detected in treated cells compared to the untreated culture, after normalization to β-actin as endogenous control. Data represent means ± S.E.M. of three independent experiments. ***P < 0.001 statistical differences in comparison to control values.

In order to assess time-dependent effects of fibrillar Aβ_1−42_ on the induction of pro-inflammatory cytokines, THP-1 cells were incubated over a 24 h period in the presence or absence of 0.5 μM Aβ_1−42_. In Aβ-treated cells, there was a rapid increase of TNF-α mRNA transcript level, that peaked at 2 h and rapidly declined by 6 h, reaching the basal levels after 16 h of incubation. The mRNA transcript levels of both IL-1β and IL-6 increased in parallel in the presence of Aβ_1−42_ and peaked at 6 h, remaining high until 24 h of incubation (Fig. 1a).

Cytokine up-regulation was a specific effect of fibrillar Aβ_1−42_, as demonstrated by the parallel treatment with other amyloid peptides that did not induce any significant changes in cytokine mRNA levels (Fig. 1b).

On the basis of these observations a period of 16 h of incubation was chosen as standard incubation time to further test the effects of BJe against Aβ_1−42_-induced pro-inflammatory response.

**Figure 2:**
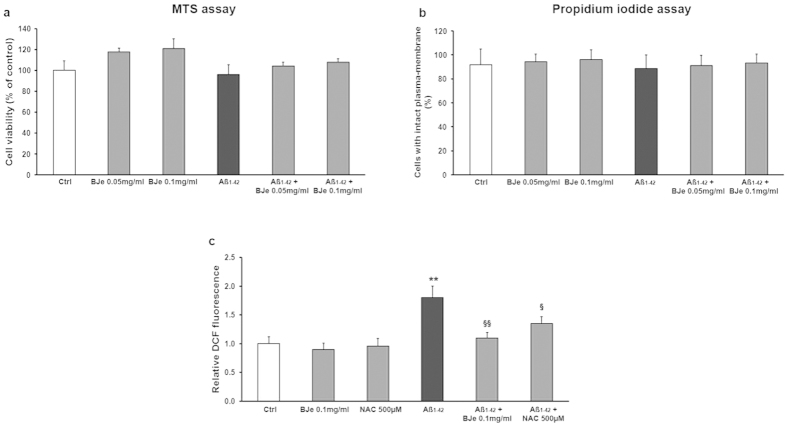
Effect of Aβ_1-42_ and BJe on THP-1 cell viability and ROS production. Cell viability was assessed by evaluation of mitochondrial activity (**a**, MTS assay) and membrane integrity (**b**, propidium iodide assay) in THP-1 cells untreated (Ctrl) or treated for 16 h with 0.5 μM Aβ_1−42_ in the presence or absence of different BJe concentrations, added to the culture medium 3 min prior to Aβ_1−42_ treatment. MTS experiments were performed in octuplicate and repeated three times; the results were expressed as percentage (mean ± S.E.M) of cell viability found in untreated cultures. PI experiments were performed in triplicate and results were expressed as percentage (mean ± S.E.M) of non fluorescent cells over the total number of cells counted. (**c**) Evaluation of intracellular ROS levels by fluorimetric measurement of oxidated DCF-DA in cells incubated with/without Aβ_1−42_ in presence or absence of BJe and NAC. **P < 0.01 statistical differences in comparison to controls. ^§^P < 0.05, ^§§^P < 0.05 statistical differences in comparison to Aβ_1−42_-treated THP-1 cells.

Under these conditions, neither significant changes in mitochondrial activity (MTS assay; Fig. 2a) nor a reduction of cell membrane integrity (PI exclusion test; Fig. 2b) were observed in THP-1 cells when 0.5 μM Aβ_1−42_ was added to cell cultures in presence or absence of different concentrations of BJe. Moreover, the incubation with BJe alone did not affect cell viability (Fig. 2a,b).

THP-1 cell treatment with Aβ_1−42_ also induced about a two fold increase of intracellular ROS levels (Fig. 2c). These effects were blunted by BJe that displayed a more powerful antioxidant effect in comparison with NAC, a well known ROS scavenger (Fig. 2c).

**Figure 3:**
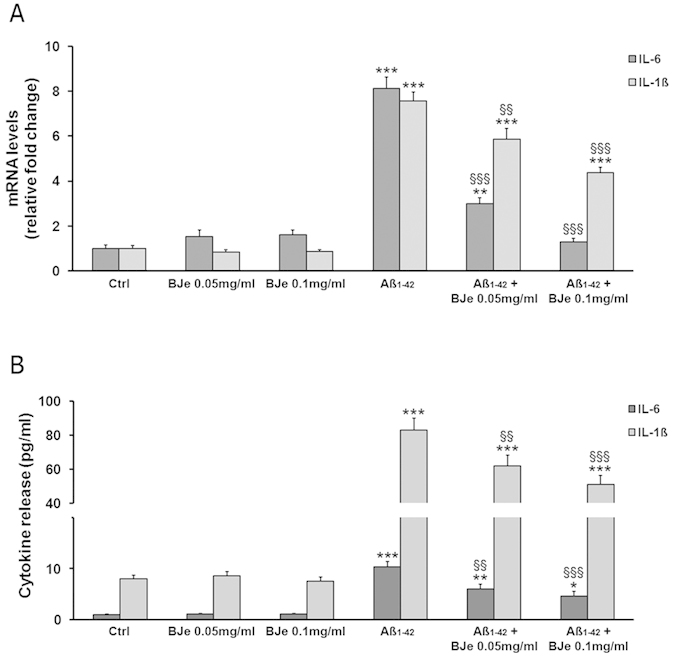
BJe reduces IL-1β and IL-6 gene expression and secretion in Aβ_1-42_-treated cells. Concentration-dependent effects of BJe against Aβ_1−42_-evoked increase in (**a**) mRNA transcript levels and (**b**) cytokine release of both IL-6 and IL-1β in THP-1 cells. Quantification of mRNA transcripts was performed by Real-time PCR and expressed as relative fold change in treated cells compared to those found in untreated culture, after normalization to β-actin. Evaluation of secreted cytokines was performed by ELISA in supernatants of THP-1 monocytes treated and untreated with BJe and Aβ_1−42_. Data are provided as pg of cytochine released *per* ml of culture media. *P < 0.05, **P < 0.01, ***P < 0.001, statistical differences in comparison to controls. ^§§^P < 0.01, ^§§§^P < 0.001, statistical differences in comparison to Aβ_1−42_-incubated THP-1 cells. Results from both Real-time PCR and ELISA experiments represent the mean ± S.E.M. of three independent experiments.

The pre-incubation with BJe was able to concentration-dependently reduce the Aβ_1−42_-induced increases in both IL-1-β and IL-6 mRNA levels; the maximal inhibitory effects were displayed by 0.1 mg/ml BJe (Fig. 3a). We also assessed the effects of Aβ_1−42_, in the presence or absence of BJe, on the secretion of both IL-1β and IL-6 after 16 h of incubation, time at which cytokine expression was still maximal. The measurements of IL-1β and IL-6 amounts released into the medium showed that Aβ_1−42_ increased the release of IL-6 and IL-1β by about 10-fold and 80-fold, respectively, into the medium. These effects were significantly reduced in the presence of BJe, that displayed the maximal effects at a concentration of 0.1 mg/ml reducing IL-6 and IL-1β concentration by about 55% and 39%, respectively (Fig. 3b).

**Figure 4:**
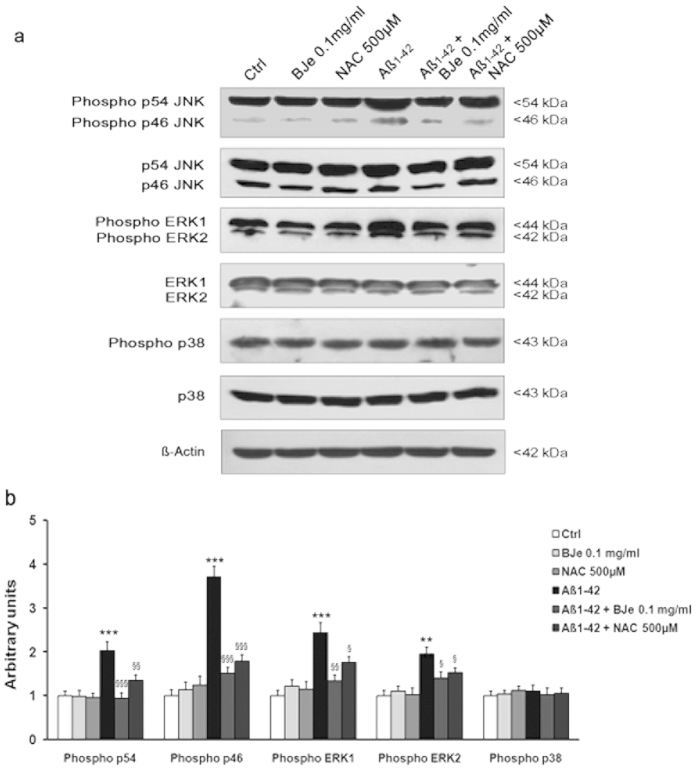
BJe reduces the phosphorylation of MAPK caused by Aβ_1-42_. (**a**) The modulation of MAPK superfamily members by BJe and NAC in THP-1 monocytes exposed to Aβ_1−42_ was evaluated by Western blot analyses, checking the phosphorylated status of MAPK proteins. Representative immunoblot images of p54, p46, p38 and ERK 1/2 are shown. The blot images were cropped around the region of interest and the samples were resolved on gels run under the same experimental conditions. (**b**) The densitometric analysis of three independent experiments (mean ± S.E.M.) is presented. **P < 0.01, ***P < 0.001, statistical differences in comparison to controls. ^§^P < 0.05, ^§§^P < 0.01, ^§§§^P < 0.001, statistical differences in comparison to Aβ_1−42_-incubated THP-1 cells.

Given that the MAPK super family of enzymes is known to regulate the inflammatory response in many cell lines, we wondered whether the Aβ_1−42_-mediated induction of a pro-inflammatory response in THP-1 cells involved the activation of MAPK members, and whether BJe was able to affect this signaling pathway. After 16 h of incubation with 0.5 μM Aβ_1–42_, we observed a 2.5 fold increase in the amounts of phosphorylated ERK 1/2 as well as p54 JNK, and a fourfold increase in those of phosphorylated p46 JNK, in THP-1 monocytes. No changes were observed in the p38 phosphorylation status under different conditions (Fig. 4a,b). The Aβ_1−42_-induced effects were counteracted when THP-1 cells were pre-incubated with BJe. In particular, the levels of phosphorylated p54 and p46 JNK were reduced by 54% and 60%, respectively, and those of phosphorylated ERK 1/2 proteins were reduced by about 45% (Fig. 4a,b). Notably, MAPK down-regulation occurred at higher extent in the presence of BJe than NAC. In fact, the reduction of protein levels achieved in the presence of NAC was 44% and 52% for phosphorylated p54 and p46 JNK, as well as 27% and 22% for phosphorylated ERK1 and ERK2, respectively (Fig. 4a,b).

**Figure 5:**
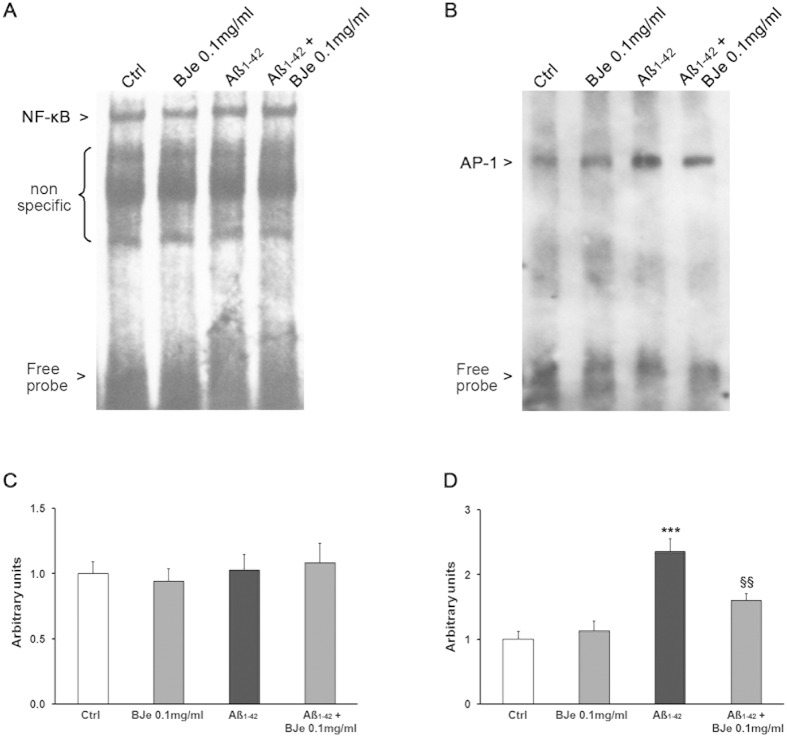
EMSA analysis of NF-kB and AP-1 in Aβ_1-42_-treated THP-1 cells after exposure to BJe. DNA binding activity of both NF-κB (**a**) and AP-1 (**b**) in nuclear extracts of untreated and Aβ_1−42_-treated THP-1 cells, in presence or absence of BJe, are shown. The densitometric analyses (**c**,**d**) of three independent EMSA assay (mean ± S.E.M.) are displayed. ***P < 0.001 statistical differences in comparison to controls. ^§§^P < 0.01 statistical differences in comparison to Aβ_1−42_-treated THP-1 cells.

We also determined whether the exposure of THP-1 cells to Aβ_1–42_ resulted in the increase of DNA binding activity by transcription factor NF-κB, given the reported involvement of NF-κB activation in cytokine up-regulation in several cell models. However, no significant differences in NF-κB activation levels were observed in THP1-cells treated with Aβ_1−42_, in the presence or absence of BJe, in comparison with controls (Fig. 5a,c). In the light of these results, we wondered whether other transcription factors could be involved in the inflammatory response evoked by Aβ_1−42_ in THP-1 cells. Therefore, given the here observed significant increase of phosphorylated ERK1/2 and p46/p54 JNK levels, we looked at the activation of AP-1, a downstream target of these MAPK members in inflammatory pathways^25^. After 16 h of incubation, we observed a 2.3-fold increase of AP-1 DNA binding activity in nuclear extracts of Aβ_1−42_-treated THP-1 monocytes (Fig. 5b–d) compared with control cells. In the presence of BJe (0.1 mg/ml), the Aβ_1−42_-induced increase of AP-1 DNA binding activity was reduced by about 35% (Fig. 5b–d).

**Figure 6:**
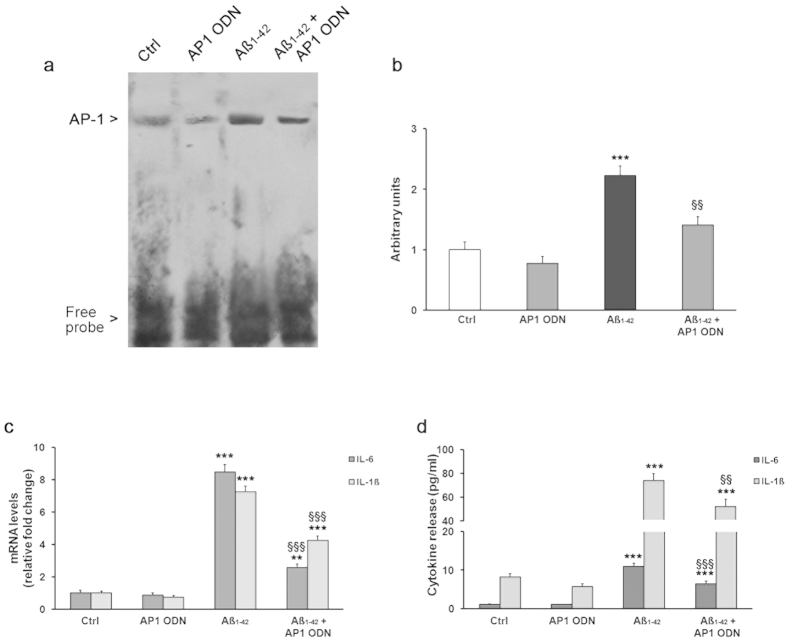
Inhibition of AP-1 DNA binding activity by AP-1 ODN attenuates Aβ_1−42_-induced up-regulation and secretion of cytokines in THP-1 cells. The effects of THP-1 cell transfection with AP-1 ODN on AP-1 activation were assessed by EMSA (**a**) in cells untreated or treated with Aβ_1−42_. Densitometric analysis of three independent blots (mean ± SEM) is reported (**b**). Evaluation of IL-6 and IL-1β production upon AP-1 ODN-mediated AP-1 inhibition was carried out by analysis of cytokine mRNA levels (**c**) and cytokine release (**d**) in THP-1 cells untreated and treated with Aβ_1−42_. **P < 0.01 and ***P < 0.001 statistical differences in comparison to controls. ^§§^P < 0.01 and ^§§§^P < 0.001 statistical differences in comparison to Aβ_1−42_-treated THP-1 cells.

Finally, to ascertain the role of AP-1 activation in inflammatory response induced by Aβ_1−42_, cell transfection with the AP-1 decoy oligodeoxynucleotide (ODN), an inhibitor of AP-1 activation, was carried out. EMSA analysis showed that treatment with AP-1 ODN significantly attenuated the AP-1 DNA-binding activity in THP-1 cells treated with Aβ_1−42_ (P < 0.01; Fig. 6a,b). In addition, the AP-1 ODN cell transfection significantly reduced increase in both gene expression and secretion of IL-1β and IL-6 induced by Aβ_1−42_ (Fig. 6c,d).

Cell transfection with either AP-1 mutant ODN or lipofectamine alone in untreated and Aβ_1−42_-treated cells did not display any significant effect on AP-1 activation (data not shown).

## Discussion

The inflammatory response to Aβ has been summarized in numerous *in vitro* models including both microglial and monocytic cells^26^,^27^,^28^,^29^. In particular, both soluble Aβ_1–40_ and fibrillar Aβ_1–42_ have been shown to mediate cellular signaling cascades involving downstream activation of tyrosine kinases, mitogen activated protein kinases (MAPKs) and transcription factors, suggesting that the activation of inflammatory pathway may be implicated in the progression of AD^30^. It has been reported that Aβ_1−42_ peptides are the first Aβ isoforms to be deposited in AD brains, and their aggregates may be considered critical for seeding plaque formation^31^,^32^. Moreover, Aβ deposition in the brain triggers a microglia-mediated inflammatory response that contributes to neuronal apoptosis and loss of memory that are characteristic of AD^33^. Indeed, the oligomeric aggregates of Aβ_1−42_ are structurally similar in size and appearance to the Aβ-derived diffusible ligands (ADDLs), which have been detected in the CSF of Alzheimer’s disease subjects^34^.

Therefore, in order to elucidate the Aβ_1−42_ effects on the activation of monocytes/macrophages, in this study we exposed the human THP-1 monocytes, an experimental model of circulating monocytes/macrophages, to Aβ_1−42_ aggregates. Notably, THP-1 monocytes have already been shown to be activated by fibrillar Aβ_1−42_ as well as the non-physiological peptide fragment Aβ_1−40_, with the result of an increase in the production of different pro-inflammatory cytokines^35^. Additionally, other observations suggest that early oligomeric Aβ_1−42_ aggregates may have a role in transforming monocytes *in vivo*^36^. These findings are relevant since it has been shown that peripheral hemopoietic cells (e.g., monocytes) are able to cross the BBB and differentiate into microglial cells in the brain parenchyma, suggesting that microglial cells arise from peripheral hemopoietic cells^37^,^38^. Indeed, the colocalization of Aβ peptide with activated microglial cells in AD brains was confirmed by histological studies^39^.

In the present study, we report that the treatment with fibrillar Aβ_1−42_ induced up-regulation and secretion of IL-6 and IL-1-β in THP-1 monocytes, two molecular events that were reduced in a concentration-dependent way by the pre-incubation with BJe. Given that monocytes/macrophages play a critical role in the inflammatory processes associated with neurodegeneration, this experimental model may represent an useful approach to evaluate the molecular mechanisms underlying the beneficial effects of BJe against neuroinflammation.

Flavonoids are widely recognized as naturally protective agents against cell damage that is also massively found in neurodegenerative conditions. Several key studies have shown that the anti-inflammatory properties of *Citrus* flavonoids are due to the inhibition of both synthesis and biological activities of different pro-inflammatory mediators, mainly the arachidonic acid derivatives, prostaglandins E2, F2 and thromboxane A2. In addition, there are well known anti-oxidant and anti-inflammatory properties of *Citrus* flavonoids that can play an important role against several degenerative pathologies including brain diseases^1^,^40^. In this regard, we observed that BJe displayed a stronger antioxidant effect than NAC, the well known ROS scavenger, against Aβ_1−42_ -induced increase of ROS levels. Recently we documented the antioxidant properties of BJe in both cell-free experimental and *in vitro* models^16^. In particular, our results showed that BJe is able to reduce both ROS generation and membrane lipid peroxidation, to improve mitochondrial functionality and to prevent DNA-oxidative damage in A549 cells incubated with H_2_O_2_^16^. The antioxidative capability of BJe was also demonstrated in an *in vivo* model of colonic inflammation^14^.

In agreement with observations by Giri and co-workers^35^, our results indicate that the up-regulation of IL-6 and IL-1β induced by fibrillar Aβ_1−42_ in THP-1 monocytes is not dependent on NF-κB activation. In this regard, it is noteworthy that both Aβ_1–40_ and Aβ_25–35,_ at higher concentrations than those used in this study, have been shown to cause NF-κB activation in THP-1 monocytes^41^. On the basis of present results, it is likely that Aβ_1−42_ is able to trigger a pro-inflammatory response in THP-1 monocytes through the activation of receptor-mediated signaling, involving kinases such as ERK and JNK, but not p38, as previously reported by Giri and co-workers^35^. We also observed that the levels of phosphorylated ERK1/2 and JNK in THP-1 incubated with Aβ_1−42_ plus BJe were significantly lower in comparison to those found in THP-1 incubated with Aβ_1−42_ alone.

Plant polyphenols in general and flavonoids in particular have long been considered effective MAPK inhibitors. Indeed, it has been shown that flavonoids, including the isoflavone genistein, limited inflammatory responses to numerous agents by inhibition of ERK phosphorylation^42^,^43^. In this study, the pre-incubation with BJe did not significantly affect the basal levels of phosphorylated MAPK proteins, but significantly inhibited Aβ_1−42_-induced MAPK activation through the reduction of phosphorylated ERK1/2 as well as JNK levels. Notably, have recently shown that BJe may inhibits MAPK phosphorylation in human colon carcinoma HT-29^11^. Moreover, it has been reported that various flavonoids are responsible for the specific inhibition of different kinases, since they have close structural homology to the PD98059, a MAPK inhibitor^44^. Inhibition of MAPK could be the results of BJe antioxidant activity, acting as a free radical scavenger in a way that is similar to how NAC works. Alternatively, it is possible that the flavonoids of BJe exert an anti-inflammatory action due to their ability to affect the activation of MAPK pathway promoted by the interaction between Aβ_1−42_ and the receptor for advanced glycation end product (RAGE). More likely, the anti-inflammatory and anti-oxidant activity of BJe is due to its ability to both destroy free radicals and interplay with specific intracellular targets involved in the neurotoxicity of Aβ_1−42_. These findings strengthen our previous observation on the potential of BJe as nutraceuticals or functional food and supplements in the context of a multitarget pharmacological strategy in the inflammation field. Previous studies showed that molecules present in BJe inhibit kinases, thus increasing the antioxidant cellular defenses mainly via the ERK/Nrf2 signaling pathway^45^. It has been reported that hesperetin modulates MAP kinase signaling by both increasing^46^,^47^ or inhibiting^48^ the phosphorylation of ERK1/2, depending by the experimental model. Also naringin is able to either inhibit^49^,^50^ or activate ERK1/2 and JNK^51^,^52^ dependently on the cell line and the experimental model used. However, a peculiarity of our study was that we used a natural extract, in which 20 flavonoids were identified^11^,^12^ that induce pharmacological effects through a network of synergies and antagonisms contributing to determine the biological effect of the phytocomplex.

Although NF-κB is commonly involved in the transcriptional up-regulation of inflammatory mediators, some results show that increases in cytokine expression may be also due to different transcription factors, such as AP-1^25^,^53^; indeed, many inflammation-related genes not only contain κB sites but also AP-1 binding sites in their promoters. Here we demonstrated that AP-1 DNA binding activity is involved in the Aβ_1−42_-induced pro-inflammatory activation of THP-1 monocytes. Notably, the incubation with BJe blocked the DNA-binding activity of AP-1, and this effect was likely associated with the significant reduction of phosphorylated JNK and ERK1/2 levels. Moreover, AP-1inhibition by AP-1 ODN was associated with a significant reduction of cytokine production in THP-1 cells exposed to Aβ_1−42_. These hallmark features collectively provide evidence for the involvement of AP-1/JNK signaling pathway in THP-1 cells stimulated by Aβ_1−42_. Therefore, the modulation of these molecular events by BJe well explains its protective effect against Aβ_1−42_-induced increases in cytokine expression and release.

Considering that monocytes play a relevant role in neurodegenerative diseases, through their activation and migration across the blood-brain barrier and their ability to initiate the inflammatory process, these data provide evidence that the BJe could be effective to reduce the inflammatory response caused by fibrillar amyloid peptides in monocytes/microglial cells. However, further investigations are needed to better understand the beneficial effects of BJe in order to develop alternative pharmacological strategies aimed at reducing the inflammatory process occurring in Alzheimer’s disease and slowing disease progression. Anyway, the ability of the main *Citrus* flavonoids to cross the blood-brain barrier^2^ makes them promising candidates for the development of general food-based neuroprotection.

## Methods

### Materials

The human leukemia monocytic cell line, THP-1, was purchased from American Type Culture Collections (ATCC; Rockville, MD, USA). RPMI-1640, L-glutamine, HEPES, sodium pyruvate, glucose, 2-mercaptoethanol, penicillin/streptomycin mixture, propidium iodide (PI), Amyloid β_1−42_ (Aβ_1−42_), Amyloid β_42−1_ reverse (Aβ_42−1_), Amyloid β_1−40_ (Aβ_1−40_), dimethylsulfoxide (DMSO), N-acetyl-cysteine (NAC), 2,7-dichlorofluorescein diacetate (DCF-DA), phosphate buffered saline solution (PBS), monoclonal antibody for β-actin, horseradish peroxidase (HRP)-conjugated anti- mouse secondary antibody, and other chemicals of analytical grade were from Sigma-Aldrich (Milan, Italy).

CellTiter 96^®^ AQueous cell proliferation kit containing the 3-(4, 5-Dimethylthiazol-2-yl)-5-(3-carboxymethoxyphenyl)-2-(4-sulfophenyl)-2H-tetrazolium (MTS) was from Promega (Milan, Italy). Fetal bovine serum (FBS), lipofectamine 2000, TRIzol , high-capacity cDNA archive kit, TaqMan Gene Expression Mastermix, TaqMan Gene Expression assays (Assays-on-Demand) for human 18S mRNA (ID: Hs99999901_s1), TNF-α (ID: Hs00174128_m1), IL-6 (ID: Hs00985639_m1), IL-1β (ID: Hs01555410_m1) were from Life Technologies (Milan, Italy). Assay location (midposition of fluorogenic probe), reference sequences and other relevant information are published online by Applied Biosystems (Foster City, CA). Instant ELISA kits for the quantitative detection of human IL-1β and IL-6 were from eBioscience (San Diego, CA, USA). MAPK and Phospho-MAPK Family Antibody Sampler Kits, containing primary and secondary antibodies to evaluate total and phosphorylated levels of p38, p44/42, and SAPK/JNK mitogen-activated protein kinases were from Cell Signaling, while the Biotin 3′-End DNA Labeling kit, LightShift Chemiluminescent Electrophoretic Mobility Shift Assay (EMSA) kit, Biodyne Nylon membranes, and Super-Signal West Pico chemiluminescent Substrate System were from Pierce Biotechnology; ECL Plus detection system and X-ray film were from Amersham Life Sciences; the above cited kits were all available from Euroclone (Milan, Italy). Developer and fixer were from Kodak (Milan, Italy).

Nuclear Protein Extraction Kit and EMSA kit for assessment of NF-κB DNA binding activity were supplied by Panomics (Diametra, Milan, Italy). Oligonucleotides containing the AP-1 consensus sequence (CGCTTGATGACTCAGCCGGAA), as well as synthetic, single-stranded phosphorothioate ODNs specific for AP-1, together with matching mutated ODN, were synthesized by MWG Biotech (Monza, Italy).

### Bergamot Juice extract

The extract of bergamot juice rich in flavonoid (BJe) has been provided by the company “Agrumaria Corleone” (Palermo, Italy) which employs bergamot fruits collected in the province of Reggio Calabria (Italy). The aqueous extract was centrifuged to eliminate impurities and then dried by the spray drying method. BJe powder was stored in small aliquots at −20 °C until use, when the drug was defrosted, diluted in culture media and filtered. The extract was previously used in other studies and its flavonoid composition has already been described^11^,^12^,^16^. The flavanones neohesperidin, naringin, hesperetin and neoeriocitrin are the molecules present in the highest amount.

### Cell culture and treatment

THP-1 cells were maintained in RPMI 1640, supplemented with L-glutamine (2 mM), HEPES (10 mM), sodium pyruvate (1 mM), glucose (2.5 g/l), 2-mercaptoethanol (0.05 mM), 10% heat-inactivated fetal bovine serum (FBS) and 1% penicillin/streptomycin, at 37 °C in a 5% CO_2_/95% air humidified atmosphere. Medium was renewed every 2 days and split performed when cells reached maximum density (1 × 10^6^ cells/ml). Before use, Aβ_1−42_ was dissolved in DMSO and kept at 37 °C for 7 days to allow fibril formation as previously described by Giri *et al.*^35^. Fibril formation was checked according to the Congo red staining method described by Wang *et al.*^54^, that is based on measurements of absorbance/turbidity at 405 nm of the Aβ sample solution. The progressive increase of absorbance of Aβ solution was an indication of the degree of aggregation that increased with the progression of aggregation.

To evaluate the time-dependent effects of Aβ on cytokine mRNA levels, THP-1 cells were seeded at a density of 5 × 10^5 ^cells/ml into culture plates and incubated at 37 °C with 0.5 μM fibrillar Aβ_1−42_, in RPMI complete medium plus 2% FBS, up to 24 h.

In further experiments, THP-1 cells were incubated at 37 °C with/without 0.5 μM fibrillar Aβ_1−42_ for 16 h, in the presence or absence of either BJe (0.05, 0.1 and 0.5 mg/ml) or NAC (500 μM), which were added to the culture medium 30 min prior to Aβ_1−42_ treatment.

To ascertain the specificity of fibrillar Aβ_1−42_ effects, in a subset of experiments soluble Aβ_1−42_ (sAβ_1−42_), Aβ_42−1_, and Aβ_1−40_, at a concentration of 0.5 μM each, were tested as controls. In all experiments, equal volumes of DMSO or PBS were added to the medium of untreated control cultures. After incubation, cells were harvested by centrifugation to assess cytokine expression, cell viability, ROS production, protein levels of MAPKs. Additionally activation of transcription factors, such as NF-κB and AP-1, was evaluated. Media were collected in order to evaluate cytokine release.

### Cell transfection using AP1 ODNs

Suspended THP-1 cells, seeded at a density of 5 × 10^5^/well into 24-well culture plates, were transfected with 1 μM double-stranded AP-1 ODN with Lipofectamine 2000, according to the manufacturer’s instructions. After 24 h of incubation, cells were exposed to 0.5 μM Aβ_1−42_ for further 16 h. Cells transfected either with mutant ODN or lipofectamine alone were used as internal negative control for transfection.

The sequences of the phosphorothioated and single-stranded decoy oligodeoxynucleotides (ODNs) were as follows: AP-1 ODN, 5′-CGCTTGATGACTCAGCCGGAA-3′, 3′-GCGAACTACTGAGTCGGCCTT-5′; corresponding mutant ODN, 5′-CGCTTGATTACTTAGCCGGAA-3′, 3′-GCGAACTAATGAATCGGCCTT-5′.

### Analysis of cytokine expression and secretion

At the end of treatments, cells were harvested by centrifugation, and total RNA was isolated using TRIzol. Then, RNA (2 μg) was reverse transcribed with High-Capacity cDNA Archive kit according to the manufacturer’s instructions. The mRNA levels of IL-6, IL-1β, and TNF-α were assessed by Real-time RT-PCR using TaqMan Gene Expression Assays, according to the manufacturer’s instructions. 18S mRNA was used as endogenous controls. Quantitative PCR reactions were set up in triplicate in a 96-well plate and were carried out in 10 μl reactions containing 1x TaqMan Gene Expression Mastermix, 1x TaqMan-specific assay, and 20 ng RNA converted into cDNA. qPCR was performed in a 7900HT Fast Real-Time PCR System with the following profile: one cycle at 50 °C for 2 min, then 95 °C for 10 min, followed by 50 cycles at 95 °C for 15 s and 60 °C for 1 min. Data were collected and analyzed using SDS 2.3 and RQ manager 1.2 software (Applied Biosystems, Life Technologies) using the 2^(−∆∆Ct)^ relative quantification method. Values are presented as fold change relative to control cells.

In order to detect human IL-6 and IL-1β, an enzyme-linked immunosorbent assay (ELISA) was performed in cell-free culture supernatants of THP-1 monocytes, using Instant ELISA Kits. Before detection, supernatants recovered from treated and untreated cells were concentrated 10-fold by freeze-drying. All freeze-dried samples were reconstituted by the addition of distilled water.

Briefly, 50 μl of standards or samples were incubated in 96-well plates at room temperature for 3 h with shaking, according to the manufacturer’s guidelines. After washing 5 times with 400 μl of wash buffer, 100 μl of the provided substrate solution were added to each well, and the plates were incubated in the dark for 10 min. Then, the enzyme reaction was stopped by adding 100 μl of stop solution into each well, and the absorbance was determined at 450 nm using a microplate reader (Tecan Italy).

### Cell viability assays

To assess either Aβ_1−42_ or BJe adverse effects on cell viability, we used both MTS and Propidium Iodide (PI) exclusion assays.

THP-1 monocytes were seeded at a density of 5 × 10^4 ^cells/well in 100 μl/well of medium without phenol red onto 96-well plates. The next day, cells were exposed to 0.5 μM fibrillar Aβ_1−42_ in presence or absence of 0.05 and 0.1 mg/ml BJe, added to the culture medium 30 min prior to Aβ_1−42_. After 16 h of treatment, 20 μl/well MTS reagents was added into each well, and the plates were incubated at 37 °C for 4 h in standard culture conditions. Then, the absorbance was recorded at 490 nm, by a microplate reader (Tecan Italia, Cologno Monzese, Italy).

PI exclusion assay was carried out, as described by Lioi and co-workers^55^ with some modifications, in order to test both cytotoxicity and membrane integrity. Briefly, 5 × 10^5^ cells were collected, resuspended in 400 μl phosphate buffered saline, and incubated with 10 μl PI labeling solution for 20 min at room temperature in the dark. The cells were then analyzed with a NovoCyte 2000 flow cytometer (ACEA Bioscences Inc., San Diego, CA, USA). A minimum of 10000 events were counted per sample.

### Measurement of Intracellular Reactive Oxygen Species

The production of ROS was quantified by fluorescent staining with DCF-DA, a non-fluorescent probe, which is oxidized to highly fluorescent compound DCF aldehyde upon exposure to ROS. At the end of treatments, cells were incubated with 5 μM DCF-DA for 30 min at 37 °C. After two washes with phosphate buffered saline (PBS) (pH 7.4), cells were centrifuged and resuspended in 500 μL of PBS supplemented with 0.1 M KH_2_PO_4_ and 0.5% Triton X-100. Cells debris were pelleted by centrifugation at 2000×g for 10 min, and the supernatants were analyzed under fluorescein optics, at an excitation wavelength of 480 nm and an emission wavelength of 540 nm. Cell lysates were analyzed for protein content using the Bradford method, and DCF fluorescence was normalized for total protein content.

### Analysis of MAPK expression

After cell lysis by extraction kit, the cytosolic fraction of cell lysates was loaded at 30 μg per well and resolved by electrophoresis on a 10% SDS-PAGE gel. Then, proteins were transferred by electroblotting onto nitrocellulose membrane, and non-specific binding sites were pre-blocked by membrane incubation with 5% non-fat dry milk in Tris-buffered saline containing 0.15% Tween 20 (TBS-T) for 1 h at room temperature. The blots were probed overnight at 4 °C with primary antibodies against total and phosphorylated JNK, ERK1/2, p38 (diluted 1:1000 in TBS-T) and β-actin (diluted 1:5000 in TBS-T); then, they were washed five times with TBS-T, and incubated for 2 h with HRP-conjugated anti-rabbit and anti-mouse secondary antibodies (diluted 1:3000 and 1:15000, respectively). After washing with TBS-T, final detection was performed by using ECL chemiluminescence system; then, bands were scanned and quantified by densitometric analysis with ImageJ 1.47.

### Assessment of transcription factor activation

At the end of incubation, THP-1 cells were harvested by centrifugation. After washing twice with cold PBS, the isolation of nuclear cell proteins was performed using a Nuclear Extraction Kit, according to the manufacturer’s guidelines. Protein concentration was determined by Bradford method. The presence of NF-κB DNA binding activity in nuclear extracts of treated and control cells was evaluated by EMSA kit according to the manufacturer’s instructions.

AP-1 binding activity was assessed by using the LightShift Chemiluminescent EMSA kit. Before analysis, the double-stranded AP-1 probe (21 bp) was biotin-end labeled by terminal deoxynucleotidyl transferase (TdT) included in the Biotin 3′ End DNA Labeling kit.

Nuclear proteins (2 μg) were incubated with the biotin-labeled NF-κB or AP-1 probes; then, the protein/DNA complexes were resolved by electrophoresis on a non-denaturing 6% polyacrylamide gel. After electroblotting onto a nylon membrane, detection of protein/DNA complexes was performed by chemiluminescence methods using streptavidin-HRP. Bands were scanned and quantified by densitometric analysis with ImageJ 1.47.

### Statistical analysis

Data obtained from three separate experiments were expressed as mean ± SEM, and analyzed by Student’s t test or one-way analysis of variance (ANOVA) followed by the post hoc Student-Newman-Keuls multiple comparisons test using GraphPad Prism (version 5.00) software (San Diego, CA). The P values lower than 0.05 were considered significant.

## Additional Information

**How to cite this article**: Currò, M. *et al.* *Citrus bergamia* Juice Extract Attenuates β-Amyloid-Induced Pro-Inflammatory Activation of THP-1 Cells Through MAPK and AP-1 Pathways. *Sci. Rep.* **6**, 20809; doi: 10.1038/srep20809 (2016).
